# Changes in spatiotemporal gait variables over time during a test of functional capacity after stroke

**DOI:** 10.1186/1743-0003-6-27

**Published:** 2009-07-14

**Authors:** Kathryn M Sibley, Ada Tang, Kara K Patterson, Dina Brooks, William E McIlroy

**Affiliations:** 1 Institute of Medical Science, University of Toronto, Toronto, Canada; 2 Department of Physical Therapy, University of Toronto, Toronto, Canada; 3 Graduate Department of Rehabilitation Science, University of Toronto, Toronto, Canada; 4 Toronto Rehabilitation Institute, Toronto, Canada; 5 Department of Kinesiology, University of Waterloo, Waterloo, Canada

## Abstract

**Background:**

Gait dysfunction and fatigue are common post-stroke, though it is unclear how extended walking activity, as would be performed during activities of daily living, may change over time. The purpose of this study was to examine if spatial and temporal gait variables deteriorate during an extended bout of walking in a test of functional capacity after stroke.

**Methods:**

24 community dwelling, independently ambulating individuals greater than 3 months after stroke performed the Six-Minute Walk Test (6MWT). Participants walked over a pressure-sensitive mat on each pass of the 30 m course which recorded spatial and temporal parameters of gait. Mean gait speed and temporal symmetry ratio during each two-minute interval of the 6MWT were examined. Additional post hoc analyses examined the incidence of rests during the 6MWT and changes in gait speed and symmetry.

**Results:**

On average, participants demonstrated a 3.4 ± 6.5 cm/s decrease in speed over time (p= 0.02). Participants who rested were also characterized by increased asymmetry in the final two minutes (p= 0.05). 30% of participants rested at some point during the test, and if a rest was taken, duration increased in the final two minutes (p= 0.001). Examination of factors which may have been associated with resting indicated that resters had poorer balance (p= 0.006) than non-resting participants.

**Conclusion:**

This study supports previous findings establishing that walking performance after stroke declines over relatively short bouts of functionally-relevant ambulation. Such changes may be associated with both cardiorespiratory and muscular fatigue mechanisms that influence performance. The findings also indicate that rest duration should be routinely quantified during the 6MWT after stroke, and consequently, further research is necessary to determine how to interpret 6MWT scores when resting occurs.

## Background

Sensorimotor control is commonly impaired following stroke, and such changes in strength and coordination can significantly affect gait [1]. Gait impairments influence functional ambulation – the capacity to perform walking during activities of daily living – and are compounded by low cardiorespiratory fitness in stroke survivors [2,3]. Furthermore, fatigue is a commonly reported issue after stroke [4,5], and cardiorespiratory and muscular components of fatigue may mutually reinforce one another. For example, both cardiorespiratory deconditioning and fibre type changes may exacerbate underlying physiological sensorimotor impairments, and ultimately compromise the functional performance of activities of daily living – in particular that of gait.

Congruent with evidence for increased fatigue and impaired gait after stroke, previous studies have demonstrated that walking speed in individuals with stroke can decrease in as little as six minutes of continuous effortful walking. Sibley et al. [6] reported that individuals in the sub-acute phase (< 3 months) after stroke covered less distance in the latter phases of the Six-Minute Walk Test (6MWT), a test of functional ambulation, compared to the initial two minutes of the test. Eng et al. [7] reported similar changes of smaller magnitude in participants in the chronic stroke phase (> 3 months) who walked even shorter distances overall. The above authors hypothesized that the changes in walking speed reflected the impact of cardiorespiratory challenge and fatigue; however, it was not possible in either of those studies to determine if sensorimotor control worsened over time and contributed to the decreased performance.

Sensorimotor control of gait may be examined through the assessment of both spatial and temporal parameters. Post-stroke reductions in gait speed [1] are well-established and provide a summary measure of the overall state of walking function. Additional indices such as the temporal symmetry ratio between paretic and non-paretic limbs can also offer further insight regarding the nature of sensorimotor impairment. Walking in healthy individuals is characterized by a symmetrical pattern between limbs, unlike post-stroke gait which is often asymmetric in timing and favors the paretic limb [8,9]. Of importance to the present study is the idea that activity-induced fatigue may influence both of these measures via cardiorespiratory (influences on speed) and peripheral muscle changes (influences on asymmetry). We hypothesize that extended periods of walking (as simulated by the 6MWT) exacerbate gait dyscontrol in individuals with stroke, and are likely linked to the associated mechanisms of fatigue.

Accordingly, the purpose of the present study was to examine changes in spatial and temporal gait parameters during an extended, effortful period of walking in individuals after stroke. We hypothesized that participants would demonstrate a progressive slowing of gait speed over the period of the test and an increased expression of gait dyscontrol, as reflected by increased temporal asymmetry. This work extends understanding of sensorimotor impairment after stroke by examining gait characteristics under the challenges that may be typical of community living (six minutes of walking) rather than very short distances (e.g. 5–10 m). This work can also provide insight into both the determinants and interpretation of indices of functional walking (such as the 6MWT) as it is applied to individuals who have had a stroke.

## Methods

This study was conducted within a larger trial on the application of a cardiac rehabilitation model post stroke. Local university and hospital research ethics committees approved the study and all participants provided informed written consent.

### Participants

Twenty-four community dwelling stroke survivors enrolled. Inclusion criteria were: ability to provide informed consent, understand the evaluation procedures, greater than three months post-stroke, have a Chedoke-McMaster Stroke Assessment (CMSA) leg impairment score greater than 2 (where voluntary movement is present without facilitation [10]), and as part of the larger trial, be able to walk at least five meters independently. Participants were excluded if they exhibited any contraindications to maximal exercise testing as outlined by the American College of Sports Medicine (ACSM) [11] or musculoskeletal impairments or pain which would limit the ability to perform the tests.

### Protocol

Participants performed the 6MWT according to standardized instructions [12]. Participants were instructed to walk as far as possible for a period of six minutes. Participants walked back and forth over a 30-meter course and performed a 180° turn at each end. They were permitted to use their walking aids and rest by standing in one location, leaning on a wall or sitting as needed. No encouragement was provided during the test. As part of the larger study, all participants had completed the test at least once before to reduce potential learning effects on 6MWT performance.

As part of the larger study, participants also completed clinical measures of stroke severity (National Institutes of Health (NIH) Stroke Scale) and sensorimotor recovery (CMSA), a maximal exercise test on a semi-recumbent cycle ergometer, a clinical balance assessment (Berg Balance Scale (BBS) [13]), and a gait assessment at preferred and fast paces. Details of the methods of these assessments are published elsewhere [3,6].

### Outcome Measures

Distance walked, rest frequency and rest duration were assessed for each two-minute interval of the 6MWT and for the entire test. Heart Rate (HR) and rating of perceived exertion (RPE, 0 – 10 scale) [14] were collected at the beginning and end of the test.

A 5 m long pressure sensitive mat was placed in the middle of the course to measure spatial and temporal gait parameters. Participants walked over the mat on each pass of the 30 m course. Gait speed and temporal symmetry ratio were averaged for each two minute interval. Temporal symmetry ratio was determined by calculating the ratio of swing time/stance time for each limb, and then dividing the limb with the larger ratio by the limb with the smaller ratio [9]. While most individuals with post-stroke asymmetry are characterized by greater stance times on the non-paretic limb, a small number of individuals appear to increase the stance time on the paretic as opposed to the non-paretic limb. As a result, it is necessary to generate an absolute ratio of symmetry with perfect symmetry equaling a ratio of 1.0 and any asymmetry increasing from 1.0, irrespective to the direction of the asymmetry.

Peak oxygen uptake (VO_2_peak), preferred gait speed and symmetry ratio, BBS, NIH, CMSA scores were extracted from the assessments completed for the larger study.

### Analysis

A one-factor, within-subjects ANOVA evaluated differences in walking distance, rest duration, gait speed and symmetry ratio between the three intervals of the 6MWT. Post hoc Tukey's tests were conducted where significant differences were observed. Preliminary analyses examining step variability measures did not show any significant changes over time. Additional analysis of patient sub-groups, comparing those who rested (REST) versus those who did not (NO REST), consisted of a two-factor ANOVA for continuous variables, chi-square tests for categorical variables and Wilcoxon-Mann-Witney test for non-parametric variables. Statistical significance was set at p < 0.05. Values expressed are mean ± standard deviation.

## Results

Clinical characteristics are presented in Table 1. Mean total distance walked was 283.3 ± 136.8 m (range 78 – 552 m). The mean increase in heart rate was 21.3 ± 14.0 beats/min (range 5 – 56 beats/min), which represented 86.8 ± 3.9% (range 49 – 115%) of peak heart rate at the end of the test and the median reported RPE score was 3 (range 1 – 10). Performance across time is illustrated in Table 2. There was a significant decrease in distance walked over time during the 6MWT [F(2, 22) = 4.3, p = 0.02]. On average, participants walked 6.4 ± 18.1 m less in the second two minutes relative to the initial two minutes, and a further 5.4 ± 17.1 m less in the final two minutes (relative to the middle two minutes). Post hoc Tukey analysis indicated that the distance walked in the final two minutes was significantly lower than that walked in the first two minutes. Spatiotemporal data for one subject was excluded due to a shuffling gait pattern which could not be analyzed by the software. There was a significant change in gait speed over time throughout the 6MWT [F(2, 22) = 4.5, p = 0.02]. This statistical difference was due to a 4.0 ± 10% decrease in speed from the first to last two minutes. In contrast, temporal symmetry ratio did not change significantly throughout the test (p = 0.5).

**Table 1:** Clinical characteristics.

	Entire cohort (n = 24)	Rest group (n = 7)	No Rest Group (n = 17)	Between-group p value
Age (years)	63 ± 13 (38 – 86)	68 ± 15 (38 – 86)	61 ± 12 (41 – 83)	0.26
Gender (M/F)	17/7	14/3	4/3	0.4
Time post stroke (months)	38 ± 26 (12 – 121)	43 ± 38 (12 – 121)	37 ± 21 (16 – 92)	0.6
Stroke type (infarct/hemorrhage/unknown)	14/8/2	5/2/0	9/6/2	0.6
Body side affected (left/right/bilateral)	11/12/1	4/3/0	7/9/1	0.7
CMSA leg	5 ± 1 (2 – 7)	5 ± 1 (2 – 6)	5 ± 1 (3 – 7)	0.8
NIH	4 ± 2 (0 – 9)	4 ± 1 (3 – 7)	/4 ± 2 (0 – 9)	0.6
Gait aid (aid/no aid)	13/11	5/2	8/9	0.3
VO_2_peak (ml/kg-min)	14.9 ± 4.6 (8.0 – 24.5)	12.5 ± 5.3 (8 – 22.5)	16.1 ± 4.0 (27.8 – 129.7)	0.1
HRpeak (beats/min)	113.3 ± 4.5 (80.0 – 148.0)	115.9 ± 5.8 (80.0 – 148.0)	108.0 ± 7.1 (90.0 – 142.0)	0.4
Preferred gait speed (cm/s)	76.2 ± 29.2 (27.8 – 129.7)	77.5 ± 25.0 (29.2 – 96.6)	75.7 ± 31.6 (0.85 – 2.46)	0.9
Preferred gait symmetry ratio	1.44 ± 0.58 (0.85 – 2.9)	1.58 ± 0.76 (14 – 52)	1.38 ± 0.5 (39–56)	0.5
Berg Balance Scale	46.7 ± 11.5 (14 – 56)	37.6 ± 15.7 (14 – 52)	51.3 ± 4.6 (39 – 56)	0.006
Total 6MWT distance (m)	283.3 ± 136.8 (55 – 552)	205.7 ± 111.3 (55 – 327)	315.2 ± 136.1 (108 – 552)	0.07
HR at end of 6MWT (beats/min)	96.3 ± 20.3 (70 – 151)	98 ± 20 (80 – 134)	95.5 ± 21.2 (70 – 151)	0.8
HR at end of 6MWT (% of HRpeak)	86.8 ± 3.9 (42 – 115)	84.1 ± 4.7 (49 – 114)	92.1 ± 7.2 (67 – 115)	0.3
RPE at end of 6MWT	4 ± 2 (1 – 10)	5 ± 1 (3 – 10)	3 ± 1 (1 – 5)	0.04

**Table 2:** Performance changes over time during the Six-Minute Walk Test.

Measure	Mean ± standard deviation		
	
	0–2 min	2–4 min	4–6 min
Distance (m)*	100.5 ± 46.1 (18 – 198)	94.1 ± 45.6 (23 – 195)	88.7 ± 49.1 (14 – 192)
Rest time** (s)	5.1 ± 11.6 (0 – 31)	8.4 ± 16.1 (0 – 44)	31.7 ± 26.4 (0 – 80)
Gait Speed (cm/s)*	92.2 ± 39.3 (23.3 – 181.9)	91.0 ± 39.1 (25.8 – 175.2)	88.7 ± 39.3 (16.6 – 172.4)
Gait Symmetry Ratio	1.48 ± 0.49 (1.04 – 2.60)	1.46 ± 0.46 (1.02 – 2.60)	1.61 ± 1.16 (1.03 – 6.67)

*p < 0.05 **For subjects who took a rest (n = 7)

### Post Hoc Analysis by Resting Status

Seven of the 24 participants rested at some point during the 6MWT, while the remaining 17 participants walked continuously. Given the high proportion of participants who rested during the test (29%), we conducted an analysis of differences between participants who rested at some point during the 6MWT (REST group, n = 7), and those who walked continuously for the entire 6MWT (NOREST group, n = 17). Within the REST group, there was a significant increase in rest duration in the final two minutes of the test [F(2, 6) = 12.1, p = 0.001]. Differences in clinical characteristics by subgroup are presented in Table 1. The BBS was significantly different between groups and was on average 14 points lower in the REST group [F(1, 19) = 9.5, p= 0.006]. All other variables were equivalent between groups, although there was a non-significant difference in VO_2_peak (3.6 ml/kg-min lower in the REST group) between groups.

Of the seven individuals in the REST group, two rested during the first two minutes, three rested in the middle two minutes, and six rested in the final two minutes. One participant rested in all three intervals, two participants rested during two intervals, and the remaining four participants rested only during one interval (which was always the final interval).

**Figure 1:**
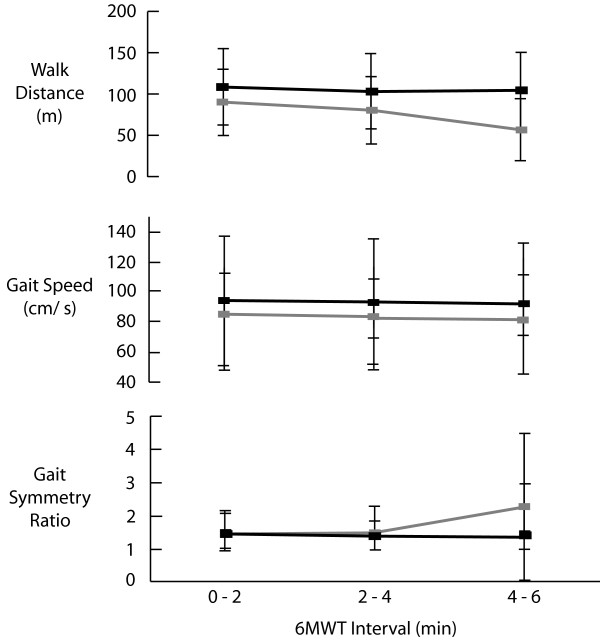
**Changes in walk distance, gait speed and symmetry over time by rest group**. Participants who walked continuously are shown in black, participants who rested in grey.

Figure 1 illustrates the changes in distance, speed and symmetry ratio over time by group. The total 6MWT distance was greater in the NOREST group (315.2 ± 136.1 m) than the REST group (205.7 ± 111.3 m), although this difference did not reach significance. HR relative to HRpeak was approximately 8% higher in the REST group at the end of the test, although this difference did not reach significance. RPE was higher in the REST group (mean 5 ± 2) than the NOREST group (mean 3 ± 1) [F(1, 19) = 6.8, p = 0.02]. There was a significant interaction for distance walked in each interval between group and time [F(2, 44) = 6.0, p = 0.005], such that distance walked in each interval was always higher in the NOREST group and REST group participants experienced greater reductions in distance walked over time. Gait speed showed a main effect of time in both groups [F(2, 42) = 3.2, p = 0.05], such that speed was significantly decreased in the final two minutes. Symmetry ratio showed a significant interaction [F(2, 42) = 3.2, p = 0.05], in which the REST group became more asymmetric in the final two minutes of the test.

## Discussion

Despite the prevalence of fatigue and well-documented reductions in functional ambulation in the stroke community, literature examining the influence of fatigue on sensorimotor control of post-stroke gait is scarce. Furthermore, there are no studies that have considered the degradation of walking induced by walking-related effort itself, neither in stroke nor in any other clinical population. There were three important observations in this study that extend on previous understanding of functional walking capacity after stroke and warrant further discussion. Firstly, we observed that gait speed, a commonly accepted measure of overall gait performance, modestly declines during the extended, albeit relatively short, bout of six minutes of walking. This decline may reveal the influence of fatigue, as reflected by perceived exertion ratings and high relative HR, on extended walking activity. Secondly, a subset of individuals demonstrated additional changes in gait symmetry over six minutes, such that gait became more asymmetrical over time. Finally, such changes in symmetry were coincident with the observation that approximately 30% of the participants could not walk continuously for six minutes and rested at some point during the test. Resting during the 6MWT has not been previously examined in the stroke literature, and has potentially important implications for the interpretation of 6MWT scores.

### Implications for declining spatial and temporal gait parameters over six minutes

The present results confirm previous reports of declines in distance walked over time during the 6MWT [6,7], and demonstrate that walking speed declines independently of walking time. In contrast, while temporal symmetry ratios did not change over time overall, lower functioning participants who rested (and covered less distance on average), did show increased asymmetry over the course of the 6MWT. Taken together, we propose that fatigue was occurring in these individuals, as reflected by change in RPE and relative HR. Such fatigue was likely occurring at both the peripheral muscle level and the cardiorespiratory system level. It is possible that change in velocity, but not gait control measures such as symmetry, may have reflected impact of cardiorespiratory fatigue, while disruptions to the control of gait (i.e. symmetry) may have reflected the additional influence of peripheral fatigue.

As there are no published guidelines for clinically significant changes in gait speed and symmetry post-stroke, we can only speculate as to the potential relevance of our findings. However, converging evidence from several populations may be used to establish a framework for consideration. For example, in Multiple Sclerosis (MS), another neurological condition with both fatigue issues and gait dysfunction, a change of 3 cm/s has been proposed to be clinically significant [15]. The mean observed 3.2 cm/s decrease in gait speed by individuals with stroke in the present study over the course of six minutes falls within this range. In addition, the stroke participants in the present study had lower preferred and 6MWT gait speeds than the MS sample, and thus were likely lower functioning. Given the lower functional status of our sample, we propose that the speed changes we observed over six minutes of walking were clinically meaningful. With respect to changes in gait symmetry, there are similarly no established standard levels of clinically meaningful change. However, participants who rested progressed from moderate to severe levels of asymmetry over the six minutes [9]. The functional implications of severe asymmetry are not yet known, though it is noted that this degree of asymmetry is associated with greater degrees of motor impairment of the leg and foot (as measured by CMSA) and is recognizable by clinical observation [9].

### Potential clinical utility for quantifying rest behavior

29% of participants in this study rested at some point during the 6MWT. The quantification of rest frequency and duration in the present study permitted the post hoc analysis of factors relating to resting behavior. 6MWT rest times have not been reported previously in the stroke literature, although resting is often cited as permitted in study protocols [16,17] as well as in the American Thoracic Society Guidelines on 6MWT administration [12]. While further study is necessary to fully examine the causes and implications of resting on the 6MWT, a number of factors warrant the recommendation that rest frequency and duration should be regularly documented during the 6MWT. Firstly, resting during the 6MWT influences the amount of walking completed per unit time, and the incidence of resting suggests a very different clinical picture. For example, two people in our sample walked a total of 327 m in six minutes, suggesting that both participants have the same 'functional capacity'. However, on closer inspection of the test results, it is revealed that Participant A walked continuously for six minutes, while Participant B rested a total of 42 seconds. Thus, despite the same outcome, the manner in which the result was achieved was very different. Quantification of rest times has implications for interpretation of 6MWT scores as well as for treatment planning. In order to more accurately interpret 6MWT scores, an additional measure of continuous walking distance (i.e. until a rest is taken) may be a valuable supplement to the traditionally used measure of total distance walked in six minutes. Identifying the number of rests taken is also important and both of these additional measures may show change over time. Clinicians can also use the occurrence of resting to identify issues in treatment planning, as an individual who rests during the 6MWT will likely also need to rest when walking in the community. In summary, there is a clear need to further examine the issue of resting during the 6MWT and revised outcome measures may be necessary to account for such variations in performance.

A second important finding related to the quantification of resting behavior links to the potential for the observation of resting to be used as a clinical prognostic indicator on the 6MWT. Specifically, we observed a difference in gait symmetry between those who rested and those who did not. While individuals who walked continuously throughout the 6MWT did not demonstrate any significant change in temporal symmetry, those who rested became severely asymmetric in the final two minutes of walking [9]. The potential link between specific control challenges influencing gait and the association to activity-related fatigue and functional capacity certainly requires further attention. In particular, when resting is observed clinicians need to consider potential dyscontrol of gait, especially after an extended period of walking. Furthermore, these findings highlight the importance of evaluating extended bouts of walking. It is not sufficient to evaluate gait merely over short distances (as is current practice).

### Determinants of resting on the 6MWT

While our findings indicate that conditions that precipitated resting in these people were also associated with increased asymmetry, we cannot determine whether the increasingly asymmetric gait pattern in individuals who rested induced the resting behavior or vice versa. Examination of factors which may have contributed to the distinction between groups suggested that BBS and potentially VO_2_peak may be linked to resting on the 6MWT. Balance has previously been identified as a very strong predictor of functional ambulation after stroke, shown to be the highest or second highest predictor of 6MWT distance among individuals with a range of abilities post-stroke [7,18-20]. Our results support this observation, and furthermore suggest that the combination of reduced balance and possibly reduced fitness was related to the resting behavior. This combination of factors may have made the 6MWT harder for people who rested. Although absolute effort as assessed by HR was equivalent between groups, individuals who rested were likely working at a higher proportion of their capacity, which was reflected by their higher RPE ratings.

There were a number of limitations to the present study. Gait could only be assessed during the middle five meters of the walking course, representing less than 20% of the total distance walked during the test. Accordingly, we are unable to speak to any changes which may have occurred outside of the pressure-sensitive mat. This includes a large segment of steady state walking as well as each end of the course which required a 180° turn. For that matter, the presence of the mat may have influenced the gait pattern itself. The mat presented a change in surface which required online adaptation and also provided participants with a visual cue that may have prompted them to focus their attention on their walking, and could have potentially ameliorated gait patterns. We did not track where participants rested, and whether rests occurred during forward walking or during turns. Ongoing development of wireless technology for both continuous cardiorespiratory monitoring [21] and real-time gait assessment using three-dimensional accelerometers will address these issues in future studies. As with all laboratory tests, there is limited external validity to real world situations, although the 6MWT parallels extended real-world walking to some degree. The sample size was relatively small and rest behavior should continue to be tracked in future studies, to both confirm these findings in individuals with stroke and examine this effect in other populations to examine whether other factors such as aging may contribute to this observation.

## Conclusion

This study presents novel findings demonstrating significant declines in gait speed during functional ambulation post stroke, additional degradation of symmetrical control in individuals who rest within the six minute assessment, and a significant prevalence of resting behavior on the 6MWT. The observation that resting behavior has a specific link to dyscontrol of gait has particular relevance for clinicians, as it could serve as a proxy indicator for breakdown of gait when quantitative assessment tools are not available. Moreover, this work re-affirms the need for appropriate rehabilitation programs, in particular for balance and cardiorespiratory fitness, post stroke, to maximize functional capacity and allow survivors to participate in meaningful activities of daily living.

## Competing interests

The authors declare that they have no competing interests.

## Authors' contributions

KMS conceived of the study, completed data collection and analysis, and wrote the manuscript. AT and KKP contributed to study design, data collection, interpretation of results, and manuscript preparation. DB and WEM contributed to study design, interpretation of results, and manuscript preparation. All authors read and approved the final manuscript.
